# Translational toxicology and exposomics for food safety risk management

**DOI:** 10.1186/1479-5876-10-S2-A41

**Published:** 2012-10-17

**Authors:** Yongning Wu

**Affiliations:** 1 China National Center for Food Safety Risk Assessment, Beijing, 100050, China

## Background

**Figure 1:**
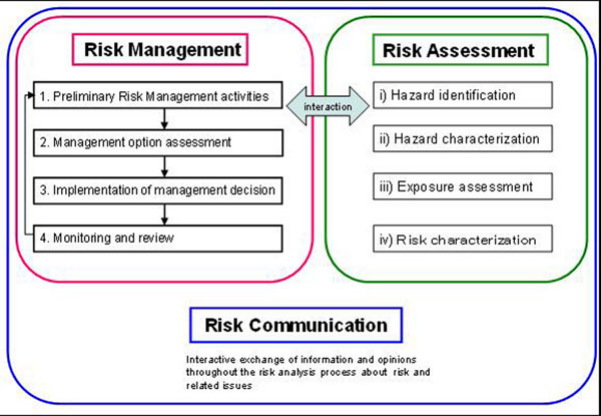
Food safety risk analysis framework

China embraces the use of risk analysis in the development of risk-based approaches for the management of public health hazards in food safety. Risk analysis is made up of three components and Figure 1 illustrates the relationship between the three components of risk analysis [1]. Estimating the magnitude and distribution of benefits and costs of particular risk management options may require addressing a myriad of concerns, e.g., changes in the availability or nutritional quality of foods; impacts on consumer confidence in the safety of the food supply or in the food regulatory system [2]. This is a brief introduction on risk management options for dealing with the new outcome from risk assessment approaches in China.

## Materials and methods

Traditionally, risk assessment is based on deterministic endpoints, i.e., use of the no observed (adverse) effect level (NO(A)EL) and the mean or high level of exposure. In the 21st century, exposure science has increasingly embraced deterministic models to predict levels of diverse exposures based on categorical data and on measured levels of pollutants in biological fluids and tissues. Increasingly, more probabilistic and distributional methods are included, to characterize the hazard(s) as well as the exposure(s). Investigations of total personal exposure initially employed external measurements of chemicals that can enter the body, which provide the more probabilistic and distributional methods. These approaches allow for more description of variability in the population as well as uncertainty in the risk estimates. Moreover, additional risk assessment outcomes are being reported, such as the margin of exposure (MOE), which gives a relative indication of the level of health concern with actually quantifying the risk [3-5].

## Results

The manner in which health reference guide values (HBGVs) such as the acceptable, tolerance, and reference dose (RfD) are estimated usually generates deterministic values in that they imply a demarcation between what is a “safe” level of exposure (i.e., exposures below the value) versus a “non-safe” level (i.e., exposures above the value). In many instances over the years, these deterministic values have been used as a common “bright line” approach to managing risk. Decision makers and competent authorities use these reference values to set standards and regulations for what are appropriate exposures. If uncertainty and variability be kept in mind, probabilistic modeling (e.g., with distributions around the values) provides risk managers more detailed dose response modeling with greater transparency of the uncertainty surrounding many of these values. To aid the decision, the risk assessment should provide information on the nature and magnitude of uncertainties in both the toxicological and exposure data that make up the inputs to the distributions being modeled.

**Figure 2:**
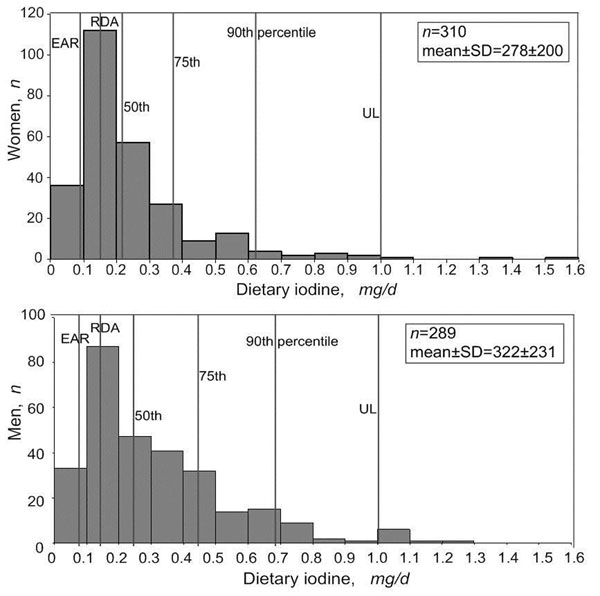
Dietary iodine intake of women and men aged 18 – 50y (excluding outliers) in four coastal provinces in China in 2009, by percentile and compared to the the Estimated Average Requirement (EAR), the Recommended Dietary Allowance (RDA) and Upper Limit (UL).

**Figure 3:**
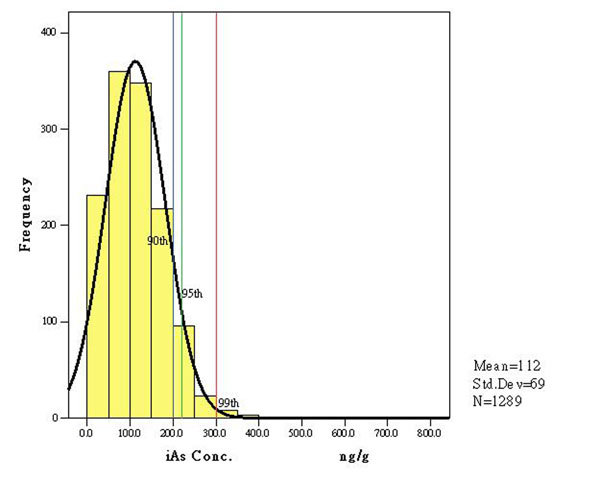
The distribution curve of inorganic arsenic concentration in overall rice samples

For risk managers, the distribution around the reference value and its probabilities and uncertainties makes decision making more complicated, particularly about who specifically or what portion of a population to protect. Considerations need to be made regarding whether the most sensitive individual(s) needs to be protected or the bulk of the general population (e.g., to decide on a goal that at least 95 % of any population should not exceed the acceptable/tolerance intake (in some cases this could be a long term goal)). For some contaminants, it may be useful to establish more than one reference value (e.g., a RfD for the general population and an acute RfD for pregnant women). The two examples will be summarized for risk-benefit analysis for universal iodized salt (figures 2) [6] and maximum limit development of inorganic arsenic in rice (table 1 and figures 3).

**Table 1:** Diet Exposure for Inorganic Arsenic (iAS) in Rice for various Cluster Diet (g/Kg.bw per day)*

Cluster Diet	A	B	C	D	E	F	G	H	I	J	K	L	M
Rice Consumption (g)	91.0	31.6	94.6	33.2	12.7	12.7	376.9	64.3	38.0	74.3	238.4	381.3	34.6

Average iAs Intake	0.17	0.06	0.17	0.06	0.02	0.02	0.69	0.12	0.07	0.14	0.44	0.70	0.06

P90 iAs Intake	0.30	0.11	0.32	0.11	0.04	0.04	1.26	0.21	0.13	0.25	0.79	1.27	0.12

P99 iAs Intake	0.46	0.16	0.47	0.17	0.06	0.06	1.88	0.32	0.19	0.37	1.19	1.91	0.17

## Conclusions

One can imagine a future in which individuals’ exposomes are contrasted between diseased and healthy populations for molecular epidemiology. In either case, the goal would be to discover causes of ill health and to generate hypotheses regarding identification and elimination or reduction of harmful exposures. These expansions of risk assessment tools and information provided require additional risk management approaches, included in the platform to translational toxicology and exposomics.

## References

[B1] Abt E Rodricks J Levy J Zeise L Burke T Science and decisions: Advancing risk assessment Risk Analysis 2010 30 1028 1036 10.1111/j.1539-6924.2010.01426.x 20497395

[B2] Carrington C PM Bolger The limits of regulatory toxicology Tox Appl Pharmacol 2010 243 191 197 10.1016/j.taap.2009.12.017 20035778

[B3] Slob W MN Pieters A probabilistic approach for deriving acceptable human intake limits and human health risks from toxicological studies: general framework Risk Analysis 1998 18 787 798 10.1111/j.1539-6924.1998.tb01121.x 9972582

[B4] Lioy P Rappaport SM Exposure Science and the Exposome: An Opportunity for Coherence in the Environmental Health Sciences Environ Health Prespect 2012 119 A466 469 10.1289/ehp.1104387 PMC3226514 22171373

[B5] Wild CP Complementing the genome with an “exposome”: the outstanding challenge of environmental exposure measurement in molecular epidemiology Cancer Epidemiol Biomarkers Prev 2005 14 8 1847 1850 10.1158/1055-9965.EPI-05-0456 16103423

[B6] Wu YN Li XW Chang SY Liu LP Zou SR Hipgrave D Variable iodine intake persists in the context of universal salt iodization in China J Nutr 2012 in press 10.3945/jn.112.157982 PMC3417834 22810983

